# Low Hartmann’s procedure or intersphincteric proctectomy for distal rectal cancer: a retrospective comparative cohort study

**DOI:** 10.1007/s00384-017-2886-5

**Published:** 2017-08-11

**Authors:** Emma Westerduin, Gijsbert D. Musters, Anna A. W. van Geloven, Marinke Westerterp, Erwin van der Harst, Willem A. Bemelman, Pieter J. Tanis

**Affiliations:** 1 Department of Surgery, Tergooi Hospital, Post box 10016, 1201DA Hilversum, The Netherlands; 2 Department of Surgery, Academic Medical Centre Amsterdam, Post box 22660, 1105AZ Amsterdam, The Netherlands; 3 Department of Surgery, Medical Centre Haaglanden, Post box 432, 2501CK The Hague, The Netherlands; 4 Department of Surgery, Maasstad Hospital, Maasstadweg 21, 3079DZ Rotterdam, The Netherlands

**Keywords:** Rectal cancer, Surgery, Hartmann’s procedure, Intersphincteric proctectomy, Complications, Pelvic abscess

## Abstract

**Purpose:**

Two non-restorative options for low rectal cancer not invading the sphincter are the low Hartmann’s procedure (LH) or intersphincteric proctectomy (IP). The aim of this study was to compare postoperative morbidity with emphasis on pelvic abscesses after LH and IP.

**Methods:**

All patients that had LH or IP for low rectal cancer were included in three centres between 2008 and 2014 in this retrospective cohort study. Follow-up was performed for at least 12 months.

**Results:**

A total of 52 patients were included: 40 LH and 12 IP. Median follow-up was 29 months (IQR 23). There were no differences between groups in gender, age and ASA classification. Seven patients in the LH group (18%) and four patients in the IP group (33%) developed a complication within 30-day postoperative with a Clavien-Dindo classification grade III or higher (*P* = 0.253). Four out of 40 patients (10%) in the LH group and two out of 12 patients (17%) in the IP group developed a pelvic abscess (*P* = 0.612). Reinterventions were performed in 11 (28%) patients in the LH group and five (42%) patients in the IP group (*P* = 0.478), with a total number of reinterventions of 13 and 20, respectively. Six and 15 interventions were related to pelvic abscesses, respectively.

**Conclusion:**

Pelvic abscesses seem to occur in a similar rate after both LH and IP. Previous reports from the literature suggesting that IP might be associated with less infectious pelvic complications compared to LH are not supported by this study, although numbers are small.

## Introduction

The surgical treatment of distal rectal cancer which does not involve the sphincter complex or pelvic floor is total mesorectal excision (TME) with or without restoration of continuity. To avoid the risks or poor function of a low anastomosis in frail elderly patients, a low Hartmann’s procedure (LH) can be performed, creating a small rectal stump and an end colostomy. Alternatively, an intersphincteric proctectomy (IP) with resection of the rectal stump and end colostomy has been proposed in these specific patients [1, 2].

If compared to IP, LH has no risk of perineal wound complications. However, LH has been associated with high rates of pelvic abscesses, especially in case of a short rectal stump (< 2 cm) [1–3]. Leaving a rectal stump could lead to stasis of rectal contents above the internal sphincter with the risk of staple line rupture and pelvic abscess formation. Persisting mucus production and diversion proctitis might result in long-term complaints of pain and discharge.

After IP, the rectum is completely resected with preservation of the pelvic floor and the perineal wound is limited. IP has been proposed to be a better solution than LH in patients who are no candidate for a coloanal anastomosis based on a high operative risk or expected poor bowel function. However, there is only little data available to conclude on the best surgical approach. Therefore, the aim of this study was to compare postoperative morbidity with emphasis on pelvic abscesses after LH and IP with a minimum follow-up of 12 months.

## Methods

### Patients

All patients from one academic medical centre (Academic Medical Centre Amsterdam) and two teaching hospitals (Tergooi Hospital Hilversum and Maasstad hospital Rotterdam) in the Netherlands who underwent a LH or IP with a permanent colostomy for primary distal rectal cancer between 2008 and 2014 were identified. Distal rectal cancer was defined as when the lower border of the tumour was within 5 cm from the anorectal junction, indicated by the upper margin of the puborectal muscle on MRI. LH or IP was considered oncologically safe by the multidisciplinary team, when considering the lower margin of the tumour. Inclusion of patients was restricted to any form of preoperative radiotherapy, the procedure being performed or supervised by a colorectal surgeon, and curative intent in order to reduce heterogeneity.

### Surgical procedures

LH consisted of an oncological rectal resection according to the TME principle, thereby creating an end colostomy and a stapled rectal remnant [5, 6]. LH could have been performed both open or laparoscopically. IP was performed using open or laparoscopic approach for the abdominal phase and with the patient either in prone position or lithotomy position for the perineal phase. Following an incision of the anoderm, the dissection was continued in the intersphincteric plane, preserving the external sphincter, levator muscles and puborectal muscle. Perineal closure was performed by layered suturing of the external sphincter and perineal skin in the midline [7, 8].

### Data extraction

Patient and treatment characteristics were retrospectively collected from patient records. Patient charts, radiology reports and operative reports were searched for patient demographics, tumour location and primary treatment characteristics. Tumour stage, circumferential resection margin (CRM), tumour perforation and lymphatic and extramural vascular invasion were extracted from the pathology report. Patient files were further searched for hospital stay, complications, reinterventions, readmissions, local recurrence, distant metastases and mortality.

### Outcome

Major postoperative complications within 30 days were defined as Clavien-Dindo grade three or higher. This includes all complications requiring surgical, endoscopic or radiological intervention (grade three), life-threatening complications requiring intensive care management (grade four) or death (grade five) [9, 10]. Pelvic abscesses and reinterventions and readmissions related to the primary surgical intervention were recorded until end of follow-up. It was decided that at least 1 year of follow-up was needed to ensure complete reporting of outcomes. If 1 year follow-up was not available in the patient files, the general practitioner or other hospitals if applicable were contacted to obtain further information regarding outcome measures. Surgical and oncological follow-up was conducted according to Dutch guidelines for rectal cancer or more frequent if necessary [4].

### Definitions

A pelvic abscess was defined as a fluid collection in the pelvic cavity as demonstrated on computed tomography (CT). Reinterventions were defined as surgical, endoscopic or radiological intervention either without anaesthesia or under local or general anaesthesia. Postoperative outcome was defined as events occurring within 30 days of surgery. Chronic presacral sinus was defined as a persistent pelvic abscess at least 1 year after surgery.

### Statistical analysis

According to distribution, numerical data were reported as median with range or interquartile range (IQR) or mean with standard deviation (SD). Categorical variables were presented as number and proportion in percentages. Comparison between groups for discrete variables was made by the Chi-square test, the Chi-square test for trend or the Fischer exact test when appropriate. The independent *t* test was used to compare normally distributed continuous variables and the Mann-Whitney *U* test was used to compare continuous variables not normally distributed. Survival rates were calculated using the Kaplan-Meier method and compared between groups using the log-rank test. *P* ≤ 0.05 was considered statistical significant. Analyses were performed using IBM SPSS Statistics for Windows (Version 23.0. Armonk, NY: IBM Corp).

## Results

A total of 52 patients were included, 34 patients from Tergooi hospital, 11 from the Maasstad Hospital and seven from the Academic Medical Centre. Forty patients were treated with LH and 12 patients with IP, all in elective setting. A total of seven different surgeons performed all procedures. Baseline characteristics are described in Table 1. A non-restorative procedure was based on a patient-based expected high risk of anastomotic leakage or poor function. In the IP group, there were significantly more low tumours than in the LH group (*P* = 0.046). The intra-operative characteristics are displayed in Table 2. The median duration of surgery was 145 min (IQR 61) in the LH group and 297 min (IQR 138) in the IP group (*P* < 0.001). There were no multivisceral resections, but three patients underwent a simultaneous procedure: hysterectomy because of uterine leiomyomas with suspicion of cancer, right hemicolectomy because of synchronous colon cancer and left adnexectomy for varicocele. Two patients with stage 4 disease underwent a synchronous resection of metastases; one patient underwent a lobectomy of the left lower lobe because of a metastasis in the lung and one patient had a metastasectomy of the liver. Pelvic drains were placed during the index procedure in 35 patients (88%) in the LH group and in eight patients (67%) in the IP group (*P* = 0.076). Significantly, more patients in the IP group underwent omentoplasty (50 vs. 13%; *P* = 0.011). Three patients had intra-operative complications. There was one patient with tumour perforation at pathological examination in the LH group. The circumferential resection margin (CRM) was at least 1 mm in all patients (Table 3).

**Table 1:** Baseline characteristics

	LHP (*n = 40*)	IP (*n = 12*)	*P* value
Sex			1.000
Male	24 (60%)	7 (58%)	
Female	16 (40%)	5 (42%)	
Age (years), mean (± SD)	74 (± 10.2)	73 (± 7.0)	0.904
BMI, median (IQR)	25.0 (8.7)	25.9 (4.5)	0.585
ASA classification			0.264
1	3 (8%)	1 (8%)	
2	22 (55%)	9 (75%)	
3	15 (38%)	2 (17%)	
Height of tumour on MRI			0.046
1 cm	1 (3%)	5 (42%)	
2 cm	9 (23%)	2 (17%)	
3 cm	11 (28%)	0	
4 cm	8 (20%)	3 (25%)	
5 cm	11 (28%)	2 (17%)	
Preoperative treatment			
Short course radiotherapy	31 (78%)	7 (58%)	0.267
Long course chemo radiotherapy	9 (23%)	5 (42%)	0.189
Indication primary colostomy			0.777
Expected high risk of leakage considering patient-related risk factors	29 (73%)	9 (75%)	
Expected poor functional outcome of ultra-low anastomosis	7 (18%)	1 (8%)	
Expected high risk of leakage related to quality of tissue	3 (8%)	1 (8%)	
Missing	1 (3%)	1 (8%)	
Timing of decision for permanent colostomy			0.287
Preoperative	23 (58%)	9 (75%)	
Intra-operative	16 (40%)	2 (17%)	
Missing	1 (3%)	1 (8%)	

*BMI* body mass index, *ASA* American Society of Anaesthesiology, *cm* centimetres

**Table 2:** Intra-operative characteristics

	LHP (*n = 40*)	IP (*n = 12*)	*P* value
Duration of surgery			
Minutes, median (IQR)	145 (61)	297 (138)	< 0.001
Technique			0.740
Open	16 (40%)	6 (50%)^a^	
Laparoscopic	24 (60%)	6 (50%)	
Multivisceral resection	0	0	1.000
Omentoplasty	5 (13%)	6 (50%)	0.011
Tumour perforation	0	0	–
Pelvic drains			0.129
No	4 (10%)	4 (33%)	
Yes, 1 drain	33 (83%)	8 (67%)	
Yes, 2 drains	2 (5%)	0	
Missing	1 (3%)	0	
Duration pelvic drainage			
Days, median (IQR)	2 (2)	9 (9)	0.006
Intra-operative complications	1 (3%)	2 (17%)	0.129
Bleeding	1	0	
Bowel injury	0	1	
Subcutaneous emphysema	0	1	

^a^In one patient laparoscopic approach was converted to an open approach because of haemodynamic instability after subcutaneous emphysema

**Table 3:** Pathology

	LHP (*n = 40*)	IP (*n = 12*)	*P* value
ypTNM tumour stage			0.804
Stage 0	4 (10%)	2 (17%)	
Stage I	13 (33%)	2 (17%)	
Stage II	9 (23%)	5 (42%)	
Stage III	13 (33%)	2 (17%)	
Stage IV	1 (3%)	1 (8%)	
Tumour perforation at pathological examination	1 (3%)	0	1.000
Positive CRM	0	0	–
Lymphatic invasion	4 (10%)	1 (8%)	1.000
Extramural vascular invasion	3 (8%)	0	1.000

### Postoperative outcome

Thirty-day postoperative major complications were observed in seven out of 40 patients (18%) in the LH group. Three patients developed a pelvic abscess within 30 days, treated by percutaneous drainage in one and transanal drainage under general anaesthesia in the two other patients. One patient had a fascial dehiscence which was operatively closed, and one patient had a bleeding from the rectal stump which was coiled. Two patients died within 30 days. Both patients developed peritonitis for which a relaparotomy was performed. One patient had a bowel perforation just below the stoma site, and one patient had a gastric perforation.

In the IP group, four out of 12 patients (33%) developed major complications, which was not significantly different from the LH group (*P* = 0.253). Transvaginal drainage of a pelvic abscess was performed under general anaesthesia in one patient and revision of a necrotic colostomy in another patient. One patient had a herniation of the appendix through a former drain opening, treated by open appendectomy. The fourth patient underwent relaparotomy for postoperative haemodynamic instability, but without the need for any intervention. There was no postoperative mortality in the IP group.

### Long-term surgical outcome

Patients were followed for a median duration of 29 months (IQR 23); 26 months (IQR 26) in the LH group and 32 months (IQR 21) in the IP group (*P* = 0.957). The proportion of patients that developed a pelvic abscess at any time until end of follow-up, including short-term postoperative outcome, was four out of 40 (10%) in the LH group and two out of 12 (17%) in the IP group (*P* = 0.612).

Overall, five patients with a drain developed a pelvic abscess, compared to one patient without drain (*P* = 1.000). All patients in the LH group who developed a pelvic abscess were drained. Date of removal of the drain was reported in one patient, and the pelvic abscess was diagnosed 12 days after drain removal. Of both patients with a pelvic abscess in the IP group, one patient received intra-operative drainage and one did not. Date of removal of the drain was reported in one patient, who developed a pelvic abscess in the presence of a pelvic drain. Duration of drainage was significantly longer in the IP group compared to the LH group (*P* = 0.006), but duration of drainage was not associated with the risk of developing a pelvic abscess (*P* = 0.539).

Of the total of four patients with a pelvic abscess in the LH group, as partially described above, one patient was treated by percutaneous drainage and three patients were treated by transanal drainage. Two of the latter underwent a second transanal drainage. In the IP group, the second patient with a pelvic abscess underwent a total of 13 endo-sponge® (B. Braun Medical B.V., Melsungen, Germany) treatments with final closure of the perineum. All abscesses were treated successfully, and none of the patients developed a chronic presacral sinus.

Complications that required reintervention occurred in 11 patients (28%) in the LH group and five (42%) in the IP group (*P* = 0.478). A total of 13 patients were readmitted at any time until end of follow-up: nine out of 40 patients (23%) in the LH group and four out of 12 (33%) in the IP group (*P* = 0.466). An overview of all reinterventions and readmissions at any time during follow-up is presented in Table 4.

**Table 4:** Postoperative outcome

	LHP (*n = 40*)	IP (*n = 12*)	*P* value
Duration of admittance			
Days, median (IQR)	15 (14)	18 (28)	0.170
Major complications within 30 days	7 (18%)	4 (33%)	0.253
Clavien-Dindo grade III	5 (13%)^b^	4 (33%)^c^	0.185
Clavien-Dindo grade IV	0	0	–
Clavien-Dindo grade V	2 (5%)	0	1.000
Pelvic abscess^a^	4 (10%)	2 (17%)	0.612
Time between surgery and diagnosis pelvic abscess			
Days, median (range)	20 (14–65)	44 (7–81)	1.000
Reintervention^a^	11 (28%)	5 (42%)	0.478
Two or more reinterventions^a^	2 (5%)	1 (8%)	0.553
Total number of reinterventions at any time during follow-up	13	20	–
Drainage of pelvic abscess	6	1	
Endo-sponge® treatment of pelvic abscess	0	13	
Closure of perineum	0	1	
Relaparotomy	3	1	
Correction of parastomal herniation	2	2	
Closure of fascial dehiscence	1	0	
Coiling of bleeding rectal stump	1	0	
Appendectomy	0	1	
Revision of necrotic colostomy	0	1	
Readmission^a^	9 (23%)	4 (33%)	0.466
Total number of readmissions	12	7	–
Pelvic abscess	5	3	
Stoma complications	3	2	
Fever	3	0	
Ileus	1	0	
Anaemia	0	1	
Herniation of appendix through drain opening	0	1	
Two or more readmissions^a^	3 (8%)	1 (8%)	1.000
Time between surgery and first readmission			
Days, median (IQR)	18 (669)	31 (88)	0.643
Total duration of readmissions until end of follow-up			
Days, median (IQR)	20 (15)	5 (15)	0.061
Total duration of readmissions due to pelvic abscess			
Days, median (range)	15 (10–29)	12 (7–16)	0.639

^a^Number of patients, at any time during follow-up

^b^Including three patients with a pelvic abscess

^c^Including one patient with a pelvic abscess

### Long-term oncological outcome

There were no local recurrences in both groups. Distant metastases were detected in six patients, all in the LH group (*P* = 0.316). The 3-year overall survival rate was 84% in the LH group and 92% in the IP group (log-rank test; *P* = 0.569).

## Discussion

This multicentre retrospective cohort study showed that there is no significant difference in major complications (Clavien-Dindo grade III or higher) or overall pelvic abscess rate between LH group and IP.

There is a great variability in literature with respect to the rate of pelvic sepsis after IP and LH. Tøttrup et al. reported that LH was associated with a 19% pelvic abscess rate, which was even 33% in the subgroup of patients with a short Hartmann stump (less than 2 cm from the pelvic floor) [2]. Sverrisson et al. reported a pelvic abscess rate of only 3% in patients undergoing LH [11]. Two other studies found a 12 and 17% pelvic abscess rate after LH, without clarification of the length of the stump [1, 3]. The variability in the rate of pelvic sepsis might be explained by a different length of the rectum stump. Possibly, the ultrashort stumps are more likely to break down. Unfortunately, in the current study, the exact length of the rectal stump in the LH group could not be reliably assessed. The distal resection margin was too inconsistently reported on by the pathologist to be able to calculate an exact length. One may assume that lower tumours will result in a shorter rectal stump, but this study did not find a difference between the height of the tumour on MRI and the development of pelvic abscesses in the LH group (*P* = 0.965).

Abdominoperineal resection (APR) has been proposed as an alternative to LH, avoiding the risk of leakage of the rectal stump. However, studies comparing LH and APR show high incidences of infectious pelvic complications for both techniques and do not conclude on superiority of any technique [1, 3, 12]. IP has the potential to reduce the perineal wound complications compared to APR by preserving the external sphincter and pelvic floor. Eriksen et al. is one of the few authors who assessed the outcome after IP with permanent colostomy as primary treatment for rectal cancer in 50 patients [7]. They reported pelvic abscesses in three patients (6%), compared to two out of 12 (17%) in the IP group of the present study.

Apart from the length of the stump, the presence of an omentoplasty could affect the incidence of pelvic abscesses. Theoretically, the omentum fills up the dead space and the well-vascularized tissue with specific immunological capacities might have a positive influence on the risk of infectious pelvic complications. Even though an omentoplasty was performed significantly more often in IP compared to LH, this did not translate into a lower pelvic abscess rate. Posthoc analysis of the BIOPEX study on pelvic closure techniques after APR did not show any impact of an omentoplasty on perineal wound healing [13]. This study neither could find any impact of placement of a pelvic drain during the index surgery on the risk of pelvic abscess formation. The expected risk of blowout or leakage of the rectal stump may be a reason for the higher number of drainages in the LH group.

A long period of follow-up beyond 30 days postoperatively is a necessity when assessing complications of pelvic surgery, since they have extensive clinical consequences resulting in multiple reinterventions and readmissions over a prolonged period of time. This especially applies to patients who received neoadjuvant radiotherapy, resembling the patients in the current series. Not only does recent literature show that these patients are at higher risk of the formation of pelvic abscesses, they are also prone to delayed healing of the abscesses with even the risk of developing a chronic pelvic sinus [14–16]. The chronic pelvic sinus is a condition which is difficult to manage with a high impact on quality of life of the patient. Chronic purulent anal or perineal discharge, pain and drains cause considerable discomfort. Abscess drainage with rinsing of the sinus sometimes requires hospital admission or specialized care at home.

The LH group had a significantly shorter duration of surgery (*P* < 0.001). The possible explanation for this difference is multifactorial. Firstly, IP is a more elaborate technique with both an abdominal and a perineal phase, whereas LH only has an abdominal phase. Secondly, when the surgeon prefers the patient to be in prone position for the perineal phase, additional time is needed to turn the patient.

Limiting factor of this study is its retrospective design, which may have resulted in incomplete data. The small sample size and few events reduce the power to find significant differences between the groups and may also lead to a type II error in the findings of similar complication rates between groups in this study. Thirdly, we have not been able to assess the correlation between the length of the rectal stump and pelvic abscess formation. Despite these limitations, we do think that our data contribute to the scarce available data on this subject. The HAPIrect collaborative study group started a randomised trial comparing LH with IP, which will hopefully bring the final answer [8].

## Conclusion

Pelvic abscesses are a significant cause for reintervention and readmissions, and this study suggests that this complication occurs in a similar rate in patients with distal rectal cancer managed by LH or IP, although numbers are small.
